# Stakeholder co-design of sustainable urban pest management strategies

**DOI:** 10.1007/s13280-025-02204-x

**Published:** 2025-07-15

**Authors:** Elizabeth C. Lowe, Nathan J. Butterworth, Alexander Austin, Cameron Webb, Tanya Latty

**Affiliations:** 1 School of Science, Edith Cowan University, 270 Joondalup Dr, Joondalup, WA 6027 Australia; 2 Cesar Australia, Brunswick, VIC 3056 Australia; 3 Department of Environment and Genetics, School of Agriculture, Biomedicine, and Environment, La Trobe University, Melbourne, VIC Australia; 4 Faculty of Science, University of Technology Sydney, Ultimo, NSW 2007 Australia; 5 Ku-Ring-Gai Council, Gordon, NSW 2072 Australia; 6 School of Medical Sciences, Faculty of Medicine and Health, The University of Sydney, Camperdown, NSW 2050 Australia; 7 Medical Entomology, NSW Health Pathology, Westmead, NSW 2145 Australia; 8 School of Life and Environmental Sciences, Faculty of Science, The University of Sydney, Camperdown, NSW 2050 Australia

**Keywords:** Cities, Integrated pest management (IPM), Invertebrates, Pest control, Practitioners, Social research

## Abstract

The high levels of broad-spectrum insecticides used to manage invertebrates in many cities around the world has significant environmental and health impacts. Integrated pest management (IPM) is a framework for sustainable pest control practice, but uptake of IPM remains low in most cities. We used participatory action research with pest management industry stakeholders (practitioners, industry representatives, researchers and government employees) to identify key issues in urban pest management in Australia, set priorities for change and discuss collaborative solutions. Via an online survey and a face-to-face workshop, the participants identified the key themes of education and training, public awareness, environmental impacts and lack of IPM uptake. We present a summary of the top eight issues, the priorities identified for each one and the co-designed recommended actions that different stakeholders can take to support a shift towards more sustainable pest management practices in urban settings.

**Supplementary Information:**

The online version contains supplementary material available at 10.1007/s13280-025-02204-x.

## Introduction

### Invertebrate pests in cities

Increasing urbanisation and global climate change is likely to result in drastic changes to urban invertebrate communities in the coming decades (Tubby and Webber 2010; Gould et al. 2018; Cuthbert et al. 2023). Land-use changes and high human population densities in cities can benefit select groups of invertebrates due to increased resources and habitat availability (Egerer and Buchholz 2021; Kotze et al. 2022), some of which can become pests, such as cockroaches and flies that are attracted to food waste, termites and ants that thrive in man-made structures, and mosquitoes and bed bugs that benefit from human hosts. Some invertebrate pests present significant direct and indirect health risks to urban residents. For example, German cockroaches transport pathogens and cause allergies (Miller and Peters 2004); mosquitoes transmit pathogens such as viruses (Brady and Hay 2020) and parasites (Nguyen-Tien et al. 2019), and termites cause billions of dollars of damage to urban buildings (Ghaly and Edwards 2011). However, cities also provide valuable habitat for a range of other invertebrates, the vast majority of which pose no risk to human health (Kotze et al. 2022), and actually improve the health of urban environments by contributing vital ecosystem services (Dangles and Casas 2019).

The definition of a pest is a species that causes damage to resources, structures or ecosystems, and/or poses a risk to human or animal health. However, the concept of a “pest” is driven by human values, and whether or not an invertebrate is considered to be a pest is often based on cultural and social perceptions, rather than actual risk of the invertebrate causing damage (Gunderman and White 2021). Similarly, in relation to pest management, decisions relating to the population densities at which the pest should be controlled (i.e. the pest management threshold) and the types of management deployed vary greatly depending on the environmental and social context. For example, in hospitality and medical industries the presence of any invertebrate can present a health risk, and therefore, all invertebrates would be considered pests that require treatment (Gliniewicz et al. 2006). In household environments, the risk presented by most invertebrates is very low, but negative public perceptions often result in all invertebrates being labelled as “pests” (Schoelitsz et al. 2019) which can increase the probability of a resident using insecticides against them (Wood et al. 1981; Gish et al. 2024).

### Insecticide use in cities

Although insecticides can be important components of pest management strategies, the excessive application of chemicals to control pests in agricultural systems causes extensive environmental and health impacts (Zhou et al. 2025). The volume and active ingredient weight of pesticides used in agriculture vastly exceeds that in urban areas—although data indicating the relative weights of pesticide use across these sectors is in fact very sparse. In many cities around the world, broad-spectrum insecticides (including pyrethroids, carbamates and organophosphates) are commonly and widely used to manage invertebrate pests in households, businesses, public spaces and civic spaces (Meftaul et al. 2020). In agricultural and natural landscapes, these chemicals have been shown to impact beneficial invertebrate species (Muratet and Fontaine 2015; Crall et al. 2018), contaminate soils and waterways (Nowell et al. 2021; Pettigrove et al. 2023; Izma et al. 2024), bioaccumulate (Alonso et al. 2012) and have detrimental effects up the food chain (Köhler and Triebskorn 2013; Yamamuro et al. 2019). Human health can also be directly affected (Alavanja et al. 2004; Sharma et al. 2020; Skidmore et al. 2023). Insecticide resistance is also now a major concern in cities (Zhu et al. 2016) and insecticide residues are widespread in urban waterways (Pettigrove et al. 2023), public housing (Julien et al. 2008) and the broader landscape (e.g. Australia’s great barrier reef (Brodie and Landos 2019)), demonstrating an urgent need to improve the sustainability of pest control practices in urban areas.

### Australia as a case study in urban pest management

Australia is one of the most highly urbanised nations, and its cities are growing at some of the fastest rates in the developed world (Australia State of the Environment 2021), providing a useful case study for mitigating challenges with increase in urbanisation, climate and pest control in cities. Pesticide use by both residents and practitioners in Australian cites is high (Pettigrove et al. 2023), and concerns about the overuse of broad-spectrum pesticides have not been widely addressed, particularly compared to other regions that have developed successful policies to reduce pesticide use (Marchand and Robin 2019). The pest control industry in Australia consists of large franchises and smaller number of local independent, often family run, small businesses (all of which must be registered). Pest control practitioners are required to complete a short course via a Registered Training Organisations to obtain their pest management technicians licence, and must keep a record of all pest management treatments (Health Pesticide regulations Australia 2011). A large range of pest management chemicals are available at local supermarkets and home maintenance stores. Residents will either undertake their own pest management or employ a practitioner, while strata organisations, civic buildings and businesses are usually managed by practitioners.

### Moving towards sustainable pest management practices

Pest management in cities needs to suppress populations of pest invertebrates, while simultaneously conserving beneficial species, including predators that contribute to natural pest control in urban landscapes (Gardiner et al. 2014). Integrated pest management (IPM) is a framework which prioritises the use of pro-active, nonchemical management practices, and strategies tailored to specific pests to minimise the impact on nontarget species and local ecosystems. Developed in the late 1950s by Smith et al. (1959), IPM is now successfully used to suppress pest populations in many agricultural systems (Maredia 2003); however, broad scale adoption of IPM has been limited in many countries (including Australia) due to lack of education, perceived risk of IPM compared to conventional chemical control methods and resistance to change (Deguine et al. 2021).

IPM can be a viable method for the management of many pests in cities (Brenner et al. 2003; Lowe et al. 2019; Gordon 2020), and would offer financial benefits for practitioners such as lower purchasing costs, access to “green” market share and better long-term outcomes for pest reduction (Siddiqi 2023). The challenge of implementing IPM is that those undertaking the pest management decisions and actions do not have the knowledge of pest biology and ecosystem ecology required to determine what types of management are most appropriate. Well-implemented pest management also requires the use of hierarchical decision-making to prioritise interventions with a low impact, only using chemicals as an absolute last resort. Promoting IPM is also a challenge because of institutional lockdown where well-established pest control networks and businesses that are heavily reliant on insecticide use, and resistant to change, prevent more sustainable options from becoming standard (Cowan and Gunby 1996). To achieve sustainable practice and facilitate the implementation of IPM in agriculture, it has been found that specific support is required to facilitate the implementation of IPM and reduce reliance on insecticides (Hillocks and Cooper 2012). In cities, it is important to have appropriate alternative pest management strategies available and to support changes in practice through legislation (Caroline 2020), but there are very few support services or incentives for either urban residents or businesses to reduce their insecticide use in Australia.

While the importance of sustainable practices and the potential of IPM to be used to manage pests in cities has been recognised in Australia (Ahmed and French 2008), uptake by residents and practitioners in urban areas has been limited (Lowe et al. 2019) and most pest control regulations do not mandate adherence to IPM principles. There is significant scope to increase IPM adoption throughout the sector. Surveys conducted overseas provide valuable insights into barriers to IPM adoption and ways to improve uptake (Deguine et al. 2021; Lane et al. 2023), but similar research has not yet been conducted in an Australian urban context.

### The role of different stakeholders in driving change

In Australia, stakeholders involved in residential urban pest management include: practitioners (independent, small and large pest control businesses), regulators (local government, state government, federal government, strata managers), the pesticide industry (chemical suppliers, business advisors, insurance providers and trainers of practitioners), researchers (entomologists, urban ecologists, urban planners) and the public (customers, landlords, homeowners, renters). Each of these stakeholder groups have varying levels of investment in sustainable pest management, different priorities in relation to the control of pests and different abilities to influence pest management policy and practice (Lowe et al. 2019).

At both a residential and commercial level, pest management is often carried out by a licenced practitioner, who is either an independent contractor or employed through a pest control business. These businesses can be small, family-owned operations or part of several larger, franchise operations. Government organisations play an important role as regulators that can influence residents behaviour related to uptake of sustainable practices around the home, and in developing legislation that provide clarity to businesses responsibilities in relation to sustainable practice (Bush 2020) which can drive investment in new methods and/or services. Local governments also have public land which requires management, including pest control (either directly or by contractors), and can decide which types of services are provided by contractors.

Industry practice and/or policy reform requires extensive stakeholder consultation to understand the issues experienced by those who manage, deliver and solicit the practices. Moreover, asking stakeholders to contribute to the decision-making processes leads to more comprehensive strategies and reforms which are more likely to gain uptake from industry (Webb et al. 2018). By understanding the needs of different stakeholders and exploring the challenges faced across these groups, it is possible to get a better understanding of how best to drive change to increase uptake of IPM (Lane et al. 2023).

### Research aims

The aim of this research is to identify the key challenges relating to implementing sustainable invertebrate pest management practices in a residential and commercial context in Australia (excluding natural areas) and to work with local stakeholders to co-design strategies to improve best practice. To do this, we conducted an online survey and a series of consultations and facilitated discussions with key pest management stakeholders to identify issues related to urban pest management. We also hope that this research will lead to a better shared understanding of the challenges faced by different urban pest management stakeholder groups, and key recommendations for a pathway to make pest management approaches in Australia more sustainable.

### Theoretical framework

To determine key sustainability challenges in urban pest management, we used a socio-ecological approach that integrated environmental factors (such as anthropogenic impacts) with social dimensions (such as how attitudes and values shape behaviour) to study complex systems and develop informed, sustainable recommendations to reduce environmental harm (de Vos et al. 2021). Our co-design approach operates on the principle that significant knowledge exists outside the academic domain, and that pathways to action that are developed with the participation of stakeholders have the best chance of success (Chevalier and Buckles 2019; Bowie et al. 2020). In addition to developing understanding of the social and environmental parameters of problems associated with complex environmental problems, the co-design process helps to build trust among stakeholders and open possibilities for collaborative solutions (Blomkamp 2018). Industry-level culture change is driven by human experience, knowledge exchange and evidence of improved practice. This is why in participatory research, the development of relationships among stakeholders and the resulting collaborative discussions are some of the most impactful outputs for driving change (Cornish et al. 2023).

We use a participatory action research method which involves working with stakeholders to apply their first-hand knowledge of a system to collaboratively develop ways to improve those systems (Cornish et al. 2023). When developing our recommendations, we use organisational change models that identify considerations for changing processes and structures within organisations and industry groups (Todnem 2005) in relation to sustainability to consider factors such as readiness for change, leadership and participation (Burnes 2017). By doing so, we ensure that the ideas generated, and the strategies developed by these stakeholders, are applicable for the different stakeholders and that our small group facilitation methods both encourage participation, ongoing observations and reflections, and have the best chance of gathering useful applied data (Honey-Rosés et al. 2020).

## Materials and methods

### Ethics

We received ethics approval from the University of Technology Sydney Human Research Ethics Committee (project reference #ETH22-7085) to collect survey information from the participants and to take written accounts of discussions made on the day of the workshop. All data were anonymised before analysis and have been stored securely.

### Selection of workshop participants

Our target participants were experienced professionals from organisations in Eastern Australia involved in the management of pests in urban areas. We recruited participants from a range of stakeholders and organisational structures within four key groups; (1) practitioners (people employed to manage pests by independent, small and large pest control business), industry (representatives from businesses that support the pest control industry such as chemical suppliers and insurance providers), government (people employed by local government and state government, including regulators) and academics (researchers from universities and research organisations).

Although the public are essential stakeholders in urban pest management through employment of practitioners and application of pesticides, it is a very diverse stakeholder group, most of whom are not engaged with issues relating to sustainability in pest management. Being able to identify sustainability challenges and design strategies for improvement requires specialist knowledge and experience within the industry, therefore, we have chosen to focus on experts who are currently working in the pest control industry.

To identify potential participants, we consulted with key industry representatives about their networks. When combined with contacts from our own networks, this resulted in an initial list of 70 contacts from 41 different organisations across Australia, mostly based in New South Wales with a small number of contacts from Victoria and Queensland. From this list, 65 invitations were sent, with five contacts discarded to avoid over representation from any one organisation. The invitation included a link to register for the workshop and the questions for the survey.

### Participant survey

As part of the workshop registration process, all participants were asked to undertake an online survey. Firstly, participants were asked to identify five issues relating to urban pest management in Australia. They were then asked “Which of the following do you think your organisation would be interested in supporting, investing in or would get value from” and were given the option to choose from the following services: integrated pest management training, pest identification training, invertebrate biodiversity surveys (i.e. in urban parks or gardens), development of IPM best practice guides, IPM advice in response to specific pest problems, public education materials relating to pest management, research into the effectiveness of IPM strategies in cities, research into the effects of pesticides on the environment and research into the effects of pesticides on human health. The options for selection were: invest or collaborate, support but not fund and/or get value from. Finally, participants were asked “Are there any other services or research topics you think your organisation would be keen to support?”.

### Participatory process to identify challenges, priorities and recommendations

Invited participants who had filled in the registration and initial survey attended a full day face-to-face workshop called “The Future of Urban Pest Control”, held at the University of Technology Sydney on the 25th of May 2022. The participants' survey responses relating to their “biggest issues in urban pest control” were grouped into themes via thematic analysis before the workshop. At the start of the workshop, six groups comprising of participants from different stakeholder groups were formed and were asked to discuss all of the themes identified in the survey, and to choose their top five issues as a group. They were then asked to discuss and note the main priorities for each of their chosen issues. Finally, groups were asked to discuss solutions that address these priorities and to identify potential areas for future collaborations. Each group had one representative from the organising committee who took notes on the discussions and the outcomes of the discussions.

### Analysis

From the survey, the relative number of responses from each of the four stakeholder groups was assessed for each theme, as well as the number of participants who indicated different levels of interest in research and services. The notes from each of the three rounds of discussions were coded into the themes, priorities and solutions chosen by participants during the workshop. We then consolidated the notes for each coded area into a series of recommendations for actions that different stakeholders can take to address the key issues and improve industry sustainability.

## Results

### Workshop participant demographics

There were 38 workshop attendees (24% practitioners, 18% researchers/academics, 34% government representatives and 24% industry representatives), primarily from the state of New South Wales (with two from Melbourne, Victoria). The participants represented 28 different organisations, including state and local government, local universities, training providers, small pest management businesses, large pest management organisations and independent contractors specialising in pest management.

### Different stakeholders’ interest in research and services

**Fig. 1:**
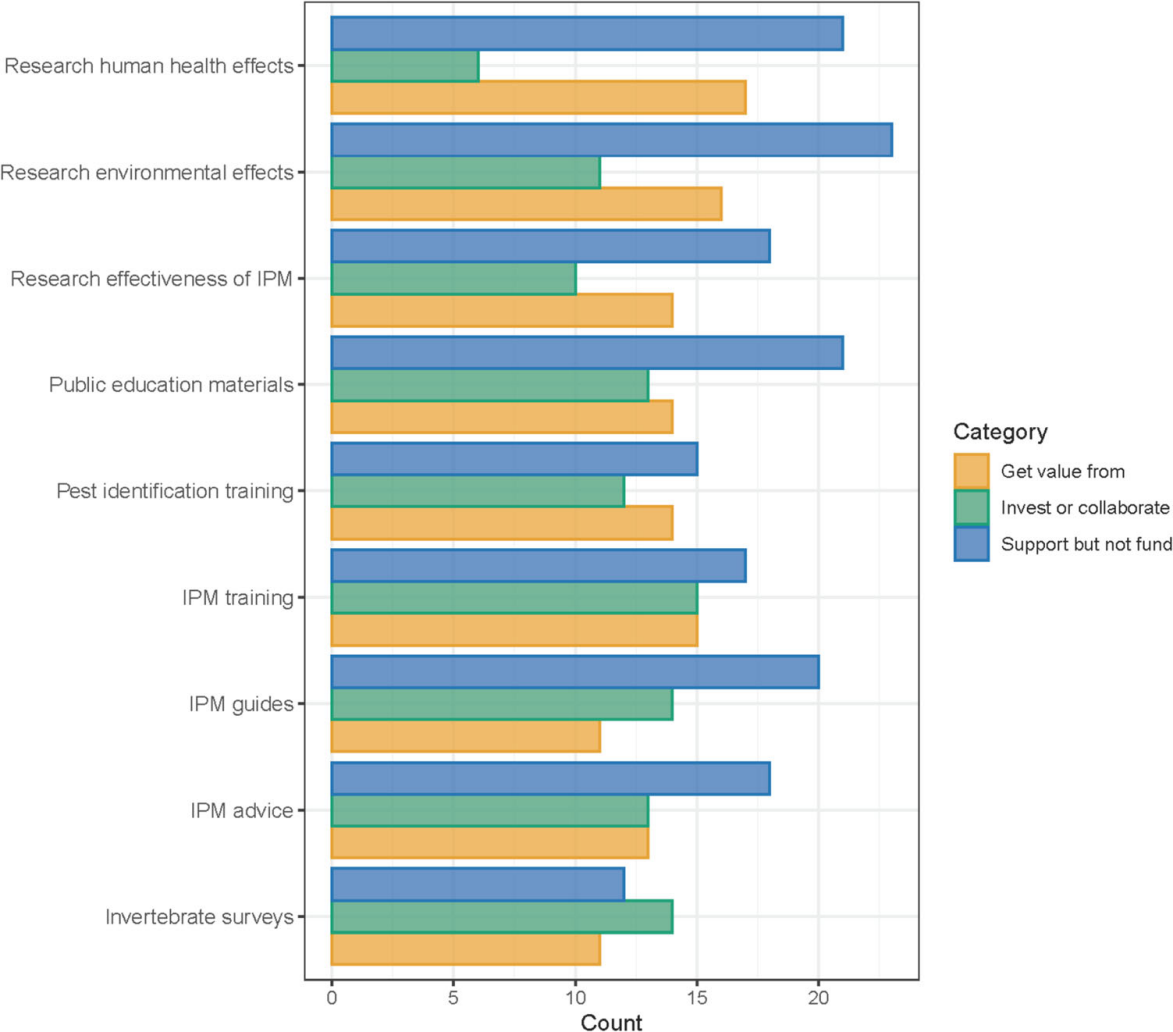
The responses when participants were asked to answer the question “Which of the following do you think your organisation would be interested in supporting, investing in or would get value from”

The survey received 37 responses, which resulted in 398 selections when asked to identify which pest management services their organisations would be interested in (Fig. 1). When identifying what their company would “get value from” the most frequently chosen options were: research into environmental effects of pesticides (*n* = 17, 46%), research into human health effects (*n* = 16, 43%) and IPM training (*n* = 15, 41%). Interestingly, although being the second ranked for value, “Research into human health effects” received the lowest score for interest in investing or collaborating, indicating that stakeholders believe this research should receive investment from outside their organisations. This is supported by the fact that the most frequently selected responses for “support (but not fund)” were: research into environmental effects of pesticides, research into human health effects and public education materials. The options which received the most responses for interest to “Invest or collaborate” were: IPM training (*n* = 15, 41%), invertebrate surveys (*n* = 14, 38%) and development of IPM guides (*n* = 14, 38%). To align with this, “invertebrate surveys” were the least frequently chosen option for “support (but not fund)”, indicating that most participants are interested in being involved in invertebrate surveys but not in funding others to do it.

When asked what other services or research the participants would be interested in, the responses included the following: sustainable pesticide disposal, pesticide lifecycle carbon emissions, pesticide bioaccumulation, the impact of all chemical pest controls (including herbicides, rodenticides and insecticides), information on chemical alternatives, development of termite baiting and barrier products, dissemination of taxonomic expertise, development of identification tools and the trajectory of urban pest infestations as climate change and urban growth increases.

### Thematic analysis of identified issues

**Fig. 2:**
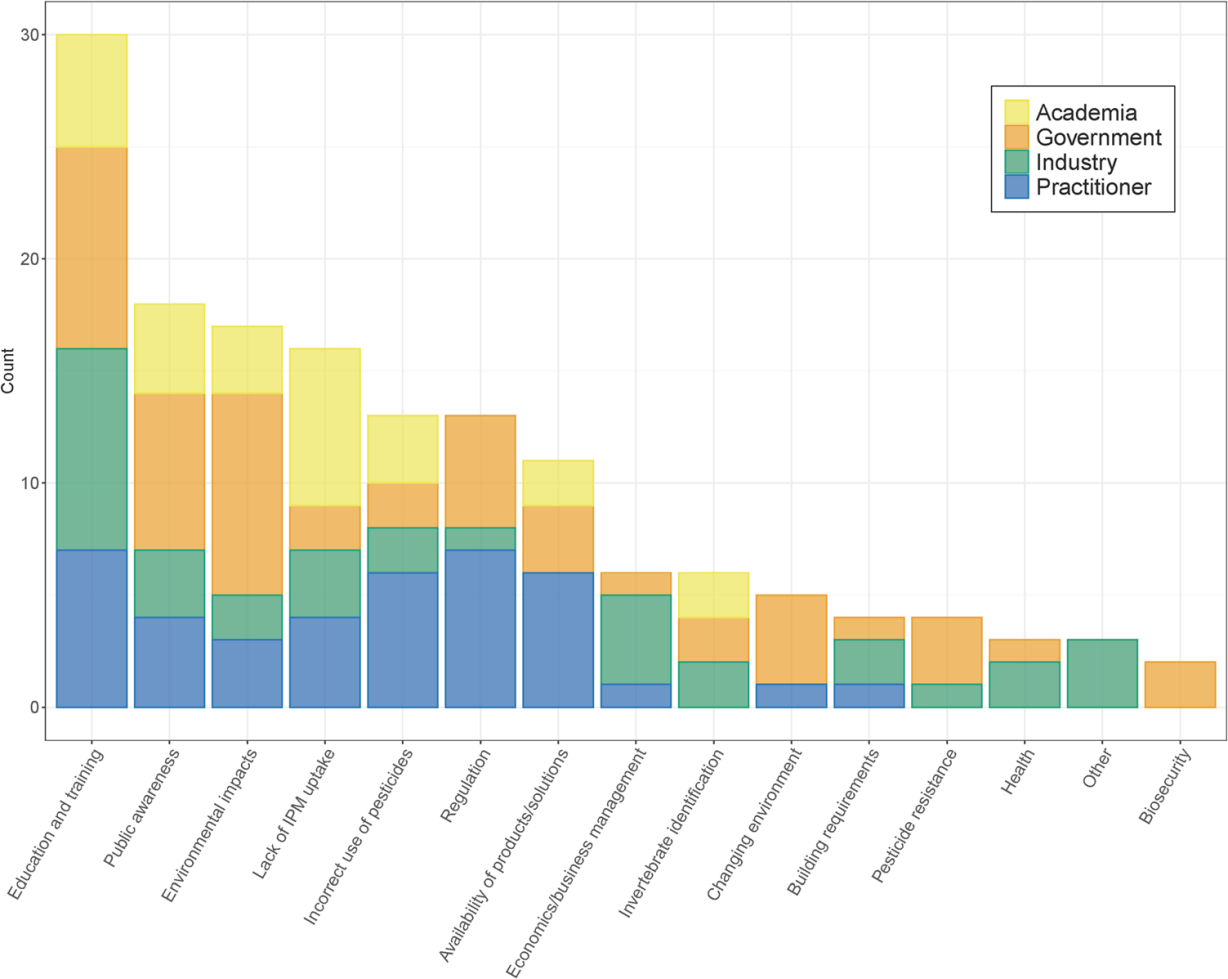
Comparison of the number of issues identified by participants from each of the four stakeholder groups (Academia, Government, Industry, Practitioner) in each of the 15 themes created from the responses

Survey participants were asked “What do you think are the 5 biggest issues in urban invertebrate pest control (Please list)” as an open-ended question. This resulted in 151 text responses identifying issues (one participant did not list any responses and some participants listed two to four responses). Each response was coded and grouped according to the topics identified, resulting in 15 distinct themes (Fig. 2). Responses included in the “other” theme were “Covid”, “Insect plagues” and “Transport”. A full list of deidentified responses can be found in supplementary Table S1.

“Education and training” was the theme with the most responses from participants, and was the category with the most responses from industry, government and practitioner stakeholder groups (Fig. 2). Examples of issues raised relating to this theme include: *“Training Courses: Outdated. Heavily tailored towards chemical application. More development towards best practices involving IPM practices”.* and *“lack of educated personnel in the industry”.* “Public awareness” was the second largest theme, with examples including: “*lack of awareness of ‘pests’ (fear of invertebrates)”,* “*Consumers being misled by industry claiming chemicals are "Safe"* and “*Customers relying on internet for information*”.

The category with the highest number of identified issues for researchers was “Lack of IPM uptake” (Fig. 2). Issues identified included: “*Strong resistance to consider or implement alternatives to conventional pest control*” and “*Most people seeking quick solutions and not seeing the big picture*”. Interestingly, practitioners were the only group of stakeholders not to list invertebrate identification as an issue. Government participants were more concerned about environmental effects, public awareness and climate change than the other stakeholders. Some of the issues raised about environmental effects included *“Pesticide contamination of waterways causing fauna mortality*”, “*Lifecycle pesticide toxicity*” and “*Lack of monitoring/information about pesticide spread in the environment*”.

Practitioners and government were the groups most concerned about lack of regulation. Examples of practitioners’ responses include *“Ease of getting a pest control licence—pest control not considered trade*” and “*Lack of policing by the EPA [Environmental Protection Agency] and other authorities on pest control companies that disobey pesticide application regulations”.* Issues raised by participants from government organisations included *“Lack of regulation of pesticides and pesticide availability and use”,* “*Enforced use of pesticides even when may not be required”* and “*Overall industry compliance with pesticides legislation”.*

### Mixed-stakeholder group consolidation of top issues

When groups composed of participants from different stakeholder groups were asked to review the 15 categories of issues and to select their top 5 “most important” issues, eight of the 15 issues were chosen by at least one group (Table 1). The two highest ranked categories were “public awareness” and “education and training” (in relation to practitioner training), which were listed as priorities by all six groups, followed by “environmental effects” which was prioritised by all except one group.

**Table 1:** The eight themes chosen as issues of main concern by the six consultative groups, the number of groups who included each issue in their top five and some key priorities identified for each issue

Theme	# of groups	Key priorities identified
Public awareness	6/6	- Ability to distinguish pest and non-pest invertebrates
		- Knowledge of best practice
		- Assessment of risk
		- Access to quality information
Education and training	6/6	- Gaps in practitioner training
		- Knowledge gaps in sustainable practice
		- Training in customer communications
Environmental effects	5/6	- Knowledge gaps of impacts
		- Lack of regulation
		- Incorrect product use
Regulation and compliance	3/6	- Accessibility of chemicals
		- Compliance and reporting
Economics and business management	2/6	- Cost of non-sustainable services
		- Client demand for non-sustainable services
		- Difficulty training staff
		- Service warranties
Invertebrate identification	2/6	- Knowledge gaps in pest identification
		- Knowledge gaps in species present in urban areas
		- Lack of motivation to identify pest for correct treatment application
Incorrect use of pesticides	2/6	- Overuse of broad-spectrum pesticides
		- Inappropriate pesticide application
		- Lack of inspections and monitoring
Building requirements	1/6	- Low uptake of pro-active protection integrated into buildings

### Priorities for each top issue

During the workshop, there were a number of structured discussions among groups of stakeholders from different organisations. Below we provide a synopsis of the discussions that were recorded for each group relating to the main priorities for each of the top eight categories (summarised in Table 1).

#### Public awareness

Lack of community knowledge of pests and unreasonable expectations of pest thresholds reduces the precision and efficiency of practice in the industry. There are many insects which are perceived as pests by the public but do not require control or are even beneficial. However, since there is demand from the public there are many companies willing to provide services that use chemicals to exterminate these insects, regardless of their lack of risk. The public requires better information on pest control “best practice” pest control, IPM strategies and alternatives to pesticides. Strategies to manage pests sustainably already exist, for example, improving hygiene and cultural control practices such as sealing cracks where pests can enter. However, lack of awareness and client demand means these methods are currently underutilised. Many practitioners lamented that even extensive explanations to clients were ineffective at convincing clients to use fewer chemicals.

There also needs to be better awareness of the impacts of over the shelf products, as there is a public perception that supermarkets can only sell “safe” products. “Greenwashing” of products as “natural” and “plant based” pesticides also means that customers and practitioners might have misinformed ideas of the safety and efficiency of different products. For example, pyrethroids are often labelled as environmentally friendly but are broad-spectrum insecticides that harm beneficial species (Mata et al. 2024). Most urban residents also have no knowledge of insecticide resistance, or the fact that overuse of products can contribute to the evolution of resistance in harmful pests (and often the ones they are trying to eradicate in their homes).

It is often unclear where the public, or businesses, should go for information on pest management. Australian local government organisations have few staff that know about invertebrates and pest control, even though they have the responsibility of managing pests from areas such as native bushland or waterways. Therefore, governments need to train their staff and provide high-quality resources on potential pest species, habitats and origins, and sustainable control methods. As governments often rely on a tender process to select contractors for pest management, guidelines on sustainable pest management practice or certifications for contractors who have training in IPM would be valuable.

#### Education and training

There is a lack of training for practitioners in IPM, pest biology, environmental impacts and drivers of pest populations. There is also a lack of understanding of the legislation/regulations around pesticide applications, which limits practitioners’ ability to apply best practice. This often results in a reactive response, dealing with problems only once they arise, rather than using monitoring programmes to prevent pest outbreaks (because these programmes require knowledge about pest biology and environmental drivers of pest emergences). There is a need for standardised IPM accreditation, ongoing professional development and professional recognition. Many pest control businesses do not prioritise staff upskilling as it does not provide an economic business case when there is no client demand for highly trained technicians. There are significant knowledge gaps on urban invertebrate management, including pest biology, insecticide resistance rates, biological control solutions and pest’s responses to IPM strategies. Better collaboration between the industry and academia is needed in order to promote research which fills these gaps and can be applied on the ground to support sustainable practice. It was also suggested that pest control practitioners need more training and support in the areas of communication and client relationships. Many practitioners may not be aware of the key role they can play in restructuring opinions on sustainable control and are rarely trained in the best ways to provide information to the clients. This means that conversations around sustainable practice and best practice do not happen.

#### Environmental effects

There is a clear need for better knowledge and awareness of environmental issues in Australian cities. Efforts such as monitoring of streams are not currently being undertaken in many areas, which means that the amounts of chemicals in the environment and the effects of low and sublethal doses of pesticides on ecosystems and public health are largely invisible. There is also no awareness about the use of targeted products or sustainable alternatives which would decrease the environmental impact. IPM can have a longer lead time to show positive results. For example, spraying a chemical may result in a lot of dead insects on the ground, while methods, such as cleaning up organic waste or emptying water in backyards, may not result in noticeable reduction in arthropod pests for many days or weeks. Many in the industry have concerns about efficiency and economic value of environmentally friendly products, which limits their use.

#### Regulation and compliance

The regulations of products with adverse environmental impacts and limiting access to dangerous chemicals need to be improved. Pesticide waste management is poorly regulated, leading to suspected health and environmental impacts. There is a high rate of change of the regulations and pesticide policies, which makes it hard for businesses and practitioners to stay up to date. Regulation in Australia is also a long way behind places like Europe and the USA. For example, fipronil is banned in Europe, but it is used extensively in Australia.

Regulations that do not distinguish between pest and beneficial invertebrates are an issue for companies that have very low thresholds for pests, for example, less than 1 insect found per week in most industrial/commercial food businesses triggers the need for chemical pest management, regardless of whether this insect is a pest or not. These thresholds are driven by import/export standards and are regulations that are very difficult to change. Pest reporting pipelines are time-consuming and there needs to be better education on the reporting process and more support from industry to adhere to reporting guidelines.

#### Economics and business management

Clients prefer cheap products and cost-effective services which undermines best practice and means that service provision decisions are based on cost, not environmental impact. The public do not recognise the value of highly trained professionals with knowledge about the pest biology and monitoring, and technicians might not get a say as to which services they provide if this is determined by consumer demand. Although the primary service of pest control is advice and consulting, customers mostly want immediate solutions, not advice for strategies that will take time and/or effort on their part. This means that it is often difficult to make a strong business case for IPM-based services in urban settings. When it comes to running a business, it is also difficult to train and retain staff, and higher levels of training are not valued.

When pest controlling companies offer a warranty it gives the client an unreasonable expectation regarding the presence of insects around their homes. It also encourages clients to call pest controllers back if they see one pest, as many practitioners will offer this as a free service for the customers while still under warranty. This is a waste of time for practitioners and may lead to the use of harsher chemicals initially to avoid return visits and associated time/financial losses.

#### Invertebrate identification

The public and, in some cases, the practitioners, are not able to differentiate between pest and beneficial insect species. Common examples are termites and ants, where many native species exist which do not present a risk, but misidentification resulting in lack of management could result in damage to the home and the pest controllers’ reputation. Without strong identification skills, practitioners are more likely to opt for systemic treatments which have a higher environmental impact and may use more of the chemical than is warranted.

Correct identification is critical for effective management actions, especially IPM-based strategies. The responsibility of insect identification lies with the pest management technician but is not currently a strength of industry training. Specific knowledge gaps include pest species biology and lifecycles, identification of less common pests, information about the actions of beneficials and pest thresholds.

#### Incorrect use of pesticides

This issue is linked to environmental impact but was classified independently because of the extensive issues relating to misuse of chemicals in the industry. Specifically, the use of broad-spectrum pesticides when not warranted, and spraying areas that do not need treatment such as lawns, fences and gardens. There are IPM methods, including application rates which reduce the amount of chemical needed, but these methods are rarely used. Another concern is the trend (and client demand) of treating routinely rather than inspecting, monitoring and responding to pest numbers.

#### Building requirements

Improvements to dwellings can be integrated into the building process or retrofitted, such as physical barriers support IPM, but are not a requirement for current building standards in Australia. There is potential for builders to make nonchemical modifications to houses to prevent the unnecessary use of pesticides; however, they are often not trained in these methods or are unwilling to take on these contracts.

## Discussion

Our overarching recommendation is that stakeholder groups are given more opportunities to work together to identify IPM strategies for specific pests, integrate research into training materials and improve regulation to support practitioner’s sustainable practice. Below we present a list of recommendations that have been developed from the workshop discussions and provide examples of actions that can be taken by different stakeholder groups.

### Support for practitioners and business owners

Our recommendations for those who deliver pest control services is to build understanding of client demand for “environmentally friendly” pest management, and to upskill in the areas of customer education and environmental impacts. Pest control businesses need better training to support clients to make decisions that prioritise nonchemical or highly selective methods and improve invertebrate identification skills using evidence-based resources. They could also improve practice by more formal monitoring of species presence and abundance to inform decision-making and evaluating long-term trends in pest occurrence to develop IPM strategies to avoid outbreaks. Practitioners would benefit from IPM decision-making tools such as those which are available for farmers (Rossi et al. 2023).

### Development of national industry standards

We recommend that practitioners work with government to develop national industry standards for IPM of different urban pests, which would improve management practice and help practitioners develop their expertise and educate their clients (Olson et al. 2018). Well-implemented education campaigns have been shown to improve public adoption of practices related to health (Wakefield et al. 2010) and sustainability (Grodzińska-Jurczak et al. 2006). This means that a consolidated campaign from the government and the pest control industry could help improve the education of pest control clients and the broader public.

### Investment in public education and communication training

Despite public awareness being identified as a priority, our survey showed that only 40% of respondents thought their company would get value from public education resources, and 57% would support the development of the resources, but not fund them. This indicates that many of the stakeholders who are responsible for implementing, researching or regulating urban pest management, do not see it as their responsibility to undertake public education. This is despite the fact that it has been shown that governments and practitioners can both play significant roles in changing client behaviour in an agricultural context (Lindén et al. 2006; Herrera et al. 2019). Therefore, we also recommend investment into training for government employees, researchers and practitioners to better communicate with the public on pest management issues. This includes being informed about ecologically sustainable alternatives to chemical use and adopting operational strategies that minimise adverse impacts on beneficial invertebrates and the broader environment. To reduce the environmental impact of pesticide applications, practitioners need to understand the consequences of different treatments and to exercise extra caution in environmentally sensitive areas or where runoff is a concern.

### More research into how IPM can be applied in cities

Researchers play a key role in understanding the use of and environmental impacts of insecticides (Meftaul et al. 2020) and developing sustainable alternatives that can be used in an urban context (Alumai et al. 2010). Social research into public engagement with the pest control industry and public attitudes towards IPM and their insecticide usage would also be valuable (Braman et al. 1998). Entomologists need to work with practitioners to test IPM strategies in different urban contexts, and to refine and validate the effectiveness of IPM. Researchers can also contribute to improving the economics of pest management by calculating the cost/benefit of IPM and identifying behaviour change strategies that reduce reliance on insecticides.

### Governments to lead by example

Government organisations play an important role in promoting invertebrate conservation and building public education and awareness through the development of resources and standards for sustainable pest control practices (Donkersley et al. 2022). This is especially the case for local government who can directly support residents to make ecologically sustainable decisions about pest control and counter misinformation coming from other sources such as the media (Mammola et al. 2022). Local governments can also demonstrate good practice by promoting and implementing IPM in public buildings and green space. State governments should introduce and mandate IPM training and accreditation schemes for practitioners, and offering incentives to businesses that demonstrate sustainable practices to increase adoption of IPM (Lefebvre et al. 2015) and support consumers to choose companies with sustainable practices. Governments can also strengthen legislation to investigate and prosecute adverse environmental effects of chemical use in urban areas, and support regulators to detect and assess environmental impacts related to pesticide use. Governments could also integrate IPM standards into building requirements, raise thresholds for non-pest and beneficial insects where appropriate, and fund the development of evidence-based standard operating procedures for different pests.

### Improve knowledge of invertebrates to help the public make informed decisions

Increasing knowledge of invertebrates for both the public and practitioners and improving attitudes towards insects around the home would help to improve sustainability of the industry via informing their pest management decisions regarding whether to control a pest and which methods/businesses to employ. For example, being able to tell the difference between pests and beneficial insects would reduce instances where pesticides are applied for non-pest species.

Increasing knowledge and changing behaviours can be done through formalised training, education campaigns and participation in citizen science, for example, participation in projects to monitor and detect pests increases participants understanding of pest issues in their local area. Citizen science projects are most effective when run with support from local government, which allows for centralised governance and the generation of long-term datasets (Conrad and Hilchey 2011); however, invertebrate citizen science activities from nongovernment organisations have also been effective at detecting pests (de Groot et al. 2023) and changing public attitudes towards insects (Sheard et al. 2024). However, these activities often only attract individuals who are already engaged with sustainability issues and do not have much influence on the attitudes of the broader public (Williams et al. 2017). Large-scale, government-funded campaigns such as those undertaken in Perth, Western Australia, to inform the public about the polyphagous shot-hole borer (Cook et al. 2023), can be used to gain broader public understanding of urban pest threats.

The public can also improve urban pest management by choosing pest control businesses with sustainable practices, improving knowledge of invertebrates and taking part in citizen science activities. Residents, who already prioritise sustainability in other aspects around the home, can be encouraged to ask potential practitioners about their approaches towards targeted pest management, nonchemical methods and reducing environmental impacts. Education will strongly influence whether the public will accept non-pest species in their gardens and use IPM (Schoelitsz et al. 2019). Therefore, residents, who are uninformed, are likely to increase demand for unnecessary services, i.e. prophylactic sprays in spring and control of invertebrates such as wasps, moths or spiders that do not present a risk to humans.

## Conclusion

The aim of this paper was to use this Australian context as a case study to explore stakeholders’ challenges relating to integrated pest management in rapidly expanding urban settings. We worked with multiple stakeholder groups to provide a shared consensus on the issues relating to sustainable pest control and to provide a pathway for improvement. Traditionally, there has been limited cooperation between practitioners and government regulators, and urban pest management has suffered from a lack of research on the challenges of pest management practice (Dhang 2011). However, in our workshop we had a high level of engagement from all participants, and very constructive conversations between stakeholder groups. An important addition to this research will be to hold similar workshops with public stakeholder groups from different areas and backgrounds to understand the attitudes that drive their pest management decisions and the best strategies to improve knowledge and engagement.

Ultimately, the details of pest management actions and the opportunities for improvement are best understood by the practitioners who undertake these actions every day, and their perspectives were vital in understanding the knowledge gaps and how to drive change. Despite the very diverse perspectives of those present at our workshop, there was a very strong desire from all present to improve best practice and to build knowledge within the industry, as well being very open to collaboration.

## Supplementary Information

Below is the link to the electronic supplementary material.

Supplementary file1 (PDF 443 KB)

## Data Availability

Data relating to this manuscript can be found in supplementary Table S1.
