# Supplementary material for: Stakeholder co-design of sustainable urban pest management strategies
Source: Ambio. 2025 Jul 15;55(1):101–14. doi: 10.1007/s13280-025-02204-x (PMC12673002; doi:10.1007/s13280-025-02204-x)
Supplement: Supplementary file 1 — Supplementary file1 (PDF 443 KB) [file 13280_2025_2204_MOESM1_ESM.pdf]

Ambio

Supplementary Information

This supplementary information has not been peer reviewed

Title: Stakeholder co-design of sustainable urban pest management strategies

Supplementary Table 1
Responses received from different stakeholder groups to the question "What do you think are the 5 biggest issues in urban invertebrate pest control (Please list)" allocated to 15 main themes.

Theme allocated	Participant stakeholder category	Issue submitted by participant
Availability of products/solutions	Academic	Integration of molecular identification techniques (i.e. EDNA)
Availability of products/solutions	Academic	eco-friendly vs. molecular/chemical control
Availability of products/solutions	Government	lack of alternatives for users
Availability of products/solutions	Government	Lack of information and availability of sustainable alternatives.
Availability of products/solutions	Government	effective treatments
Availability of products/solutions	Practitioner	Humane pest control
Availability of products/solutions	Practitioner	DIY pest control products
Availability of products/solutions	Practitioner	German Cockroach control
Availability of products/solutions	Practitioner	Fermentation Fly control/education
Availability of products/solutions	Practitioner	External house fly management in residential situations.
Availability of products/solutions	Practitioner	Subterranean termite nest detection or location
Biosecurity	Government	Biosecurity threats
Biosecurity	Government	new and emerging pests
Building requirements	Government	urban design
Building requirements	Industry	Building Practices
Building requirements	Industry	Pre-construction termite management systems
Building requirements	Practitioner	Recruiting builders and handy people to make non-chemical changes to prevent the unnecessary use of pesticides. Builders don't like doing small jobs.
Changing environment	Government	climate change
Changing environment	Government	changing urban landscape
Changing environment	Government	Climate Change
Changing environment	Government	climate change
Changing environment	Practitioner	global warming
Economics/ Pest control market	Government	economic losses
Economics/ Pest control market	Industry	Staffing

Economics/ Pest control market	Industry	Market penetration - too few people use pest controllers
Economics/ Pest control market	Industry	Consolidation of the market
Economics/ Pest control market	Industry	Merge of big pest control companies
Economics/ Pest control market	Practitioner	Heavy Influenced by Chemical Manufacturers (conflict of interest):
Environmental effects	Academic	Use of pesticides that are harmful to the environment and beneficial organisms
Environmental effects	Academic	decline of biodiversity
Environmental effects	Academic	pollution of environment
Environmental effects	Government	environmental persistance
Environmental effects	Government	impact on surrounding wildlife/invertebrates
Environmental effects	Government	risk of harm to non target species
Environmental effects	Government	Pesticide contamination of waterways causing fauna mortality
Environmental effects	Government	Loss of biodiversity due to pesticide use
Environmental effects	Government	Lack of monitoring/information about pesticide spread in the environment, including lack of understanding of how pesticides are mobilised by stormwater.
Environmental effects	Government	habitat management
Environmental effects	Government	issues around waste in/around urban areas
Environmental effects	Government	The impact of insecticides on native urban wildlife.
Environmental effects	Industry	knowledge of the potential environmental impacts of insecticides
Environmental effects	Industry	secondary poisoning
Environmental effects	Practitioner	Lifecycle pesticide toxicity
Environmental effects	Practitioner	Current pest control practices that involve outdoor spraying, particularly of lawns, fences, gardens etc. and the effect that has on beneficial and non-target invertebrates, which would also include run-off
Environmental effects	Practitioner	Consideration of impact of pesticides on the urban environment.
Health	Government	Human health impacts
Health	Industry	threat to human and environmental health
Health	Industry	Safety
Incorrect use of pesticides	Academic	Excess use of pesticides at premisses (house/commercial)
Incorrect use of pesticides	Academic	Be conscious about the application of pesticides in green areas
Incorrect use of pesticides	Academic	Broad application of pesticides without specific target group
Incorrect use of pesticides	Government	Insecticide misuse

Incorrect use of pesticides	Government	inappropriate use of pesticides in residential settings
Incorrect use of pesticides	Industry	Application
Incorrect use of pesticides	Industry	Pest Managers treating routinely rather than inspecting and asking questions
Incorrect use of pesticides	Practitioner	pesticide overapplication
Incorrect use of pesticides	Practitioner	broad-spectrum pesticide use
Incorrect use of pesticides	Practitioner	poor application practices that result in drift/ run-off into nearby ecosystems
Incorrect use of pesticides	Practitioner	overuse of pesticides
Incorrect use of pesticides	Practitioner	unsafe use of pesticides
Incorrect use of pesticides	Practitioner	Managing spray drift created during external spider treatments in residential situations
Insect ID	Academic	species identification
Insect ID	Academic	Accurate species identification
Insect ID	Government	identification of good and bad invertebrates (they are not all bad)
Insect ID	Government	pest identification
Insect ID	Industry	Identification and knowing habits of pests
Insect ID	Industry	Not able to identify pests less commonly found
Lack of IPM uptake	Academic	access to unbiased services
Lack of IPM uptake	Academic	managing and maintaining desirable invertebrates while eliminating pests
Lack of IPM uptake	Academic	To provide information about alternatives mechanism to avoid pests inside the houses.
Lack of IPM uptake	Academic	To provide biological information about the animals to the customers
Lack of IPM uptake	Academic	wrong intention
Lack of IPM uptake	Academic	Most people seeking quick solutions and not seeing the big picture
Lack of IPM uptake	Academic	Strong resistance to consider or implement alternatives to conventional pest control
Lack of IPM uptake	Government	Over reliance on chemical agents
Lack of IPM uptake	Government	a lack of professional pest controllers with an awareness of and willingness to use alternative controls.
Lack of IPM uptake	Industry	New technologies and practices can encounter resistance
Lack of IPM uptake	Industry	Integrated Pest Management
Lack of IPM uptake	Industry	IPM
Lack of IPM uptake	Practitioner	The broad-scale use of pesticides as a perceived "easy fix" to pest problems.
Lack of IPM uptake	Practitioner	Integrated pest management is not using a variety of different chemicals.
Lack of IPM uptake	Practitioner	Overuse of pesticides - Lack of implementing IPM principles

Lack of IPM uptake	Practitioner	A willingness to integrate less impacting control measures, many IPM's may only pay lip service to the environment.
Other	Industry	Current Covid issues
Other	Industry	Insect plagues
Other	Industry	Transport
Pesticide resistance	Government	overuse leading to immunity
Pesticide resistance	Government	pesticide resistance
Pesticide resistance	Government	Insecticide resistance
Pesticide resistance	Industry	insecticide resistance
Public awareness	Academic	public naivety of insects
Public awareness	Academic	Unawareness
Public awareness	Academic	People's lack of empathy for insect, spiders and other invertebrates
Public awareness	Academic	General human disconnection with the rest of nature
Public awareness	Government	lack of awareness of impacts of chemicals
Public awareness	Government	lack of awareness of 'pests' (fear of invertebrates)
Public awareness	Government	public awareness
Public awareness	Government	Public perception
Public awareness	Government	education & awareness
Public awareness	Government	public understanding/expectation
Public awareness	Government	pesticide awareness of the general public
Public awareness	Industry	Public perception
Public awareness	Industry	Customers relying on internet for information
Public awareness	Industry	Bites by 'invisible' pests
Public awareness	Practitioner	Lack of knowledge and desire by the public on how to encourage urban gardens that increase biodiversity
Public awareness	Practitioner	Fear and/or lack of tolerance of spiders and other invertebrates by home owners, that drives the demand for outdoor spraying
Public awareness	Practitioner	Intolerance to creatures. One creature does not equate to an infestation.
Public awareness	Practitioner	Consumers being misled by industry claiming chemicals are "Safe" "Natural" "Organic" etc. These representations should be banned by law to be used in the industry.
Regulation	Government	unregulated environmental contamination
Regulation	Government	Monitoring data and surveillance
Regulation	Government	Lack of regulation of pesticides and pesticide availability and use. Enforced use of pesticides e.g. termiticides as part of DAs etc, even when may not be required.

Regulation	Government	Overall industry compliance with pesticides legislation
Regulation	Government	effective regulation with resources available
Regulation	Industry	supporting government policies
Regulation	Practitioner	Lack of policing by the EPA and other authorities on pest control companies that disobey pesticide application regulations and good practices
Regulation	Practitioner	The too narrow definition of sensitive sites and required notification. All urban areas should be considered sensitive!
Regulation	Practitioner	Ease of getting a pest control licence - pest control not considered trade under ANZCO
Regulation	Practitioner	unregulated use of pesticides
Regulation	Practitioner	Many rodenticides are too easily accessible to the general public with little to no instruction.
Regulation	Practitioner	No/Poor Regulatory Policing by EPA.
Regulation	Practitioner	Any homeowner (without a licence) can buy ANY chemical from Any supplier and on-line for DIY application. This is not policed/regulated.
Training	Academic	Chemical awareness
Training	Academic	pest training of practitioners
Training	Academic	To provide more information to customers about pesticides contamination during application
Training	Academic	no education in sustainable pest control
Training	Academic	Research on alternatives unappreciated and underfunded
Training	Government	understanding threshold levels
Training	Government	Future leaders in the industry (technical managers were traditionally entomologists, but this is no longer offered at the undergraduate level)
Training	Government	Training
Training	Government	Understanding the impacts of pest control decisions
Training	Government	understanding the drivers for pest control decisions.
Training	Government	personal initiatives
Training	Government	funding, resource
Training	Government	maintaining appropriate training and skills of EPA licensed practitioners
Training	Government	education
Training	Industry	Lack of technical training
Training	Industry	understanding of IPM/inability to troubleshoot difficult situations
Training	Industry	Training
Training	Industry	Training
Training	Industry	Knowledge
Training	Industry	Training

Training	Industry	Funding for research on major urban pests
Training	Industry	Lack of educated personnel in the industry
Training	Industry	Training/certification
Training	Practitioner	termite/construction interface knowledge
Training	Practitioner	Lack of knowledge and understanding of insect identification and ecology.
Training	Practitioner	Inconsistent quality of RTO training outcomes
Training	Practitioner	inadequate training
Training	Practitioner	Training and Knowledge in control measures.
Training	Practitioner	Accessing that information.
Training	Practitioner	Training Courses: Outdated. Heavily tailored towards chemical application. More development towards best practices involving IPM practices.
